# Prediction of functional properties of nano $\hbox {TiO}_2$ coated cotton composites by artificial neural network

**DOI:** 10.1038/s41598-021-91733-y

**Published:** 2021-06-10

**Authors:** Nesrine Amor, Muhammad Tayyab Noman, Michal Petru

**Affiliations:** Department of Machinery Construction, Institute for Nanomaterials, Advanced Technologies and Innovation (CXI), Technical University of Liberec, Studentská 1402/2, 461 17 Liberec 1, Czech Republic

**Keywords:** Nanoscale materials, Theory and computation, Information technology, Software

## Abstract

This paper represents the efficiency of machine learning tool, i.e., artificial neural network (ANN), for the prediction of functional properties of nano titanium dioxide coated cotton composites. A comparative analysis was performed between the predicted results of ANN, multiple linear regression (MLR) and experimental results. ANN was applied to map out the complex input-output conditions to predict the optimal results. A backpropagation ANN model called a multilayer perceptron (MLP), trained with Bayesian regularization were used in this study. The amount of chemicals and reaction time were selected as input variables and the amount of titanium dioxide coated on cotton, self-cleaning efficiency, antimicrobial efficiency and ultraviolet protection factor were analysed as output results. The accuracy of the proposed algorithm was evaluated and compared with MLR results. The obtained results reveal that MLP provides efficient results that are statistically significant in the prediction of functional properties ($$p<0.1 e^{-10} $$) compared to MLR. The correlation coefficient of MLP model ($$>95\%$$) indicates that there is a strong correlation between the measured and predicted functional properties with a trivial mean absolute error and root mean square errors values. MLP model is suitable for the functional properties and can be used for the investigation of other properties of nano coated fabrics.

## Introduction

Titanium dioxide ($\hbox {TiO}_2$) in nano forms (nanoparticles, nanowires, nanorods, nanosheets, nanoflowers) have shown tremendous impact in many industries (especially in textiles) as a multifunctional coating material. Nano $\hbox {TiO}_2$ is frequently used to achieve not only photocatalytic self-cleaning, antimicrobial properties, superhydrophilic surfaces and water purification due to its higher surface area, but also in protective textiles against ultraviolet (UV) radiations, noise mitigation and air pollution^1,2^. $\hbox {TiO}_2$ is an intrinsic n type metal oxide semiconductor material that closely resembles with zinc oxide in photocatalytic properties. The prominent features that enables $\hbox {TiO}_2$ as a functional material in multiple applications are photocatalytic activity, chemical stability and non-toxicity^3^. Researchers have synthesized and coated nano $\hbox {TiO}_2$ on textile substrates for photocatalytic and other functional properties^4–7^. In an experimental study, Noman et al. synthesized $\hbox {TiO}_2$ nanoparticles and successfully coated on cotton fabric. The coated fabric was analysed against methylene blue dye and bacteria culture for self cleaning and antimicrobial properties respectively. The coated fabric showed significant results for self-cleaning and antimicrobial properties. The design of experiment was based on central composite design (CCD) and the obtained results were statistically evaluated under regression model through Design Expert (DE) software^8^. Here, in this current study, an attempt has been made to develop a prediction model by using machine learning tools that can work in two ways i.e., correlates the actual response of coated fabric with process variables, analyse the predicted response of DE and indicate which approach is better as a prediction model in reality. Nowadays, ANN exhibits a strong advantage in capturing any type of existing relationship from given data as it does not include a physical mechanism and a mathematical model. Thanks to the training process, ANN can learn, understand and recognize the information treatment rules, adapt and predict the wanted output variables from database considered as input variables^9^.

In general, textile processes are mostly non-linear in nature and a lot of efforts are applied to obtain optimal solutions^10–12^. ANN is an excellent approach that has been widely used by different group of researchers for the prediction of various properties of textile materials for different purposes where it has proven its effectiveness and potential. Malik et al. applied a backpropagation ANN to predict the tensile properties of even and uneven yarns extracted from polyester-cotton blend. The selected parameters for this study were twist multiplier, cot hardness and break draft ratio. Simulation results of tensile properties were obtained through ANN and compared with MLR, where ANN outperformed MLR^13^. In another study, Malik et al. proposed ANN for the prediction the warp and weft yarns crimp in woven barrier fabrics. The use of ANN to predict yarn crimp has shown good results in the predicted output, especially for the warp yarn (with less prediction error between actual and predicted output)^14^. In another experimental study, Malik et al. used ANN for the prediction of antimicrobial performance of chitosan/AgCl-$\hbox {TiO}_2$ coated fabrics. The input variables were curing time and concentration of colloids. Samples were developed with different blends of selected colloid under different curing time. Backpropagation ANN was trained under a hybrid combination of Bayesian regularization and Levenberg Marqaurdt algorithms^15^. The same group of Malik et al. extended their study and applied ANN to develop a relationship between loom parameters, used material and construction of fabric in terms of porosity, mean pore flow, mean pore size with air permeability. The experimental result showed ANN provided prediction with high accuracy of comfort properties with minimal error^16^. Almetwally et al. applied ANN and linear regression for the prediction of core spun yarn strength, elongation and rupture. The results showed that ANN propose highly accurate prediction of spinning strength^17^. Farook et al. used ANN to predict cotton fibre maturity. They selected various fibre characteristics as an input variables and analyzed fibre maturity as an output variable. The simulation results showed that ANN predicted cotton fibre maturity with small error^18^. In another study, Farooq et al. proposed ANN to predict the change of shade of dyed knitted fabrics that would happen after finishing application. The inputs were the shade percentage, dye color, and finishing concentrations. The outputs were the delta values of the selected samples with respect to standard samples. Tests results showed that ANN provide high prediction accuracy for shade change that occurred during finishing^19^. Dashti et al. predicted the yarn tenacity using ANN trained by genetic algorithm. The performance of this approach was useful to achieve desired tenacity with minimum production cost. However, it is a time-consuming process^20^. Furferi et al. introduced ANN for the prediction of coatings process on textile fabrics. Testing results demonstrated the significance of ANN model particularly for coating mechanisms^21^. Knanat et al. applied ANN for the prediction of thermal resistance of wet knitted fabrics. The results showed efficient prediction of thermal resistance^22^. Ribeiro et al. proposed an automated machine learning method to predict physical properties of woven fabrics based on finishing features and textile design^23^. However, the prediction of overall mechanical behavior of textile composites is still a very challenging task due to the complexities of microstructures and boundary conditions^24,25^. Taieb et al. used ANN for the prediction of fabric drapability under low stress^26^. They reported that physical factors play crucial role while predicting fabric drapability properties. Kalkanci et al. estimated fabric shrinkage by applying ANN algorithm inside relaxation methods^27^. Thermofixing, sanforizing, drying and washing are the important processes that are applied on fabrics during finishing applications^28^. Dimensional changes were predicted at the end of finishing processes by ANN. The experimental results showed that ANN gives better prediction results for dimensional change. Khan et al. investigated mechanical properties of cross-ply laminated fibre-reinforced polymer composites as well as modelled and predicted the mechanical properties using ANN^29^. The composite samples were developed by altering glass fibre layers with carbon fibre layers and polyphenylene sulphide with high-density polyethylene. The fibers were used as reinforcement materials and polyphenylene sulphide was used as a polymer matrix. Mechanical properties i.e., hardness, flexural modulus, impact and rupture strength were investigated for both directions. Simulation results showed that ANN predicts the mechanical properties with low MAE which computed between actual and predicted values.

The above discussed literature reveal that the most common ANN type used in textile industry is MLP^30,31^. MLP is a class of backpropagation ANN that has the advantages of self-learning, high nonlinearity resolution and the ability of mapping between input and output variables without introducing a mathematical model between nonlinear data and precisely predict the best function. As well as the authors searched, there is no relevant literature available on the use of ANN in any form to investigate or predict the functional properties of nano $\hbox {TiO}_2$ coated textiles. Therefore, an approach has been introduced in this paper that is based on MLP model for the prediction of various properties of nano $\hbox {TiO}_2$ coated cotton. The amount of titanium precursor, amount of solvent and process time were selected as input variables whereas the amount of nano $\hbox {TiO}_2$ coated on cotton fabric, and some related functional properties i.e., self-cleaning efficiency, antimicrobial efficiency and ultraviolet protection factor (UPF) were considered as outputs variables. The achieved results were compared with MLR and with the experimental values using analysis of variance (ANOVA).

## Materials and methods

### Materials

Bleached cotton fabric with mass (GSM) 110 g m$^{-2}$ was used for samples preparation. All other chemicals i.e., titanium tetrachloride, isopropanol and methylene blue dye were taken from sigma aldrich.

### Experimental design

Design of experiment with various amount of titanium tetrachloride and isopropanol under fluctuating sonication time is based on central composite design for all developed samples as shown in Table 1. The optimization of independent variables was performed by Design-Expert 10 software. Total 20 samples were developed during experimental study. The variables used during the study were the amount of titanium precursor (titanium tetrachloride), the amount of solvent (isopropanol) and sonication time. The combination of variables is expressed in Table 1. The experimental results were obtained by using the following quadratic Equation.

1 $$\begin{aligned} Y =b_0+\sum b_i X_i + \sum b_{i,j} X_i X_j + \sum b_{i,i} {X_i}^2, \; where \; i\ge j \; and \; i,j=1,2,3. \end{aligned}$$

In the above equation, $b_0$ represents the coefficient of constant term, $b_i$ represents the coefficient of linear term that explains the persuade of the variables, $b_{i,j}$ represents the coefficient of two factors interaction and $b_{i,i}$ represents the coefficient of quadratic term respectively^8^.

**Table 1:** The input variables and experimental design.

Sample	Amount of titanium tetrachloride (ml)	Amount of isopropanol (ml)	Sonication time (h)
1	10	6	0.5
2	6	4	3
3	2	2	0.5
4	6	4	4
5	6	2	3
6	10	2	4
7	2	6	4
8	6	6	2
9	10	6	2
10	6	4	1
11	6	4	2
12	2	4	1
13	6	4	3
14	6	4	1
15	2	6	0.5
16	2	2	4
17	6	4	2
18	10	4	3
19	6	4	1
20	10	2	0.5

### Artificial neural network

Artificial intelligence techniques seem to be promising and are still evolving. ANN are machine learning algorithms based on mathematical models that are identical and inspired by biological nerve systems, responsible for the functionality of human brain. It consists of large sets of neurons connected by axons. Artificial neurons are individual neural units that are interconnected with each other to form a network.The crucial point of this technology is the connection between individual neural units that can be reinforcing or inhibiting. This action is exercised through a combination of input values and an activation function, which returns the output of a neuron. A very special feature of ANN is the automatically creation, derivation and exploration of new information using previous learning that is called as training process^32^.

A multilayer perceptron method is a class of feedforward ANN. Backpropagation (BP) is one of the most common and typical learning algorithm in MLP that deals with non-linear models by reducing the desired target error in a gradient descent pattern though tailoring the weight factors and biases^33,34^. In this algorithm, training occurs in three steps: (1) Forward propagation step: an experimental data is introduced to MLP as input and its effect is propagated, in stages, through different layers of the network. Then, as a result, the outputs are generated. (2) Computation of the error: the error vector is computed from the difference between predicted and actual outputs. (3) Backward propagation step: The computed error vector is propagated backwards to the MLP and the synaptic weights are adjusted in such a way that the error vector reduces with every iterative step. Furthermore, the MLP model is getting closer and closer to generating the desired output.

**Figure 1:**
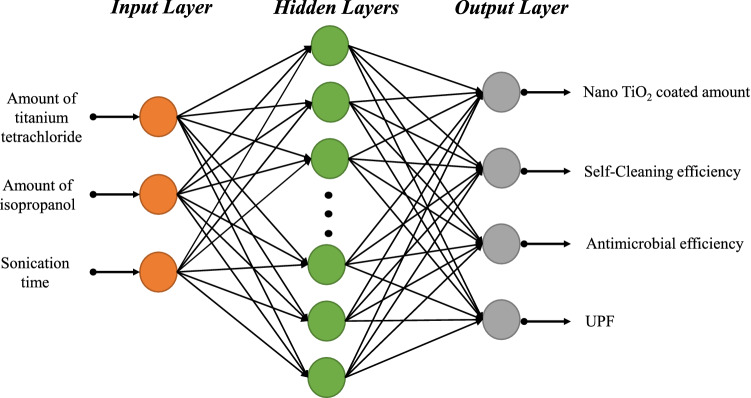
MLP model for the prediction of functional properties of nano $\hbox {TiO}_2$ coated cotton.

Technically, MLP are used to model non-linear problems in order to predict output dependent variables $y= [y_{1}, \ldots , y_{n}]$ for given independent input variables $x= [x_{1}, \ldots , x_{k}]$ from their training values. The obtained results mainly depend on weights $w= [w_{1}, \ldots , w_{k}]$. The following equation represents the relationship between input and output of the network^35,36^:

2 $$\begin{aligned} y =\varphi \left( \sum _{j} w_j *x_j +b \right) , \end{aligned}$$

where, *y* is the output. $x_j$ is the *j*th input. $w_j$ is the *j*th weight and *b* represents the bias. $\varphi$ is the activation function. The biases and weights comprise the information that the neuron recovers during the training phase. A detail theoretical discussion of ANN architecture and training algorithms are presented by different researchers in their studies^9,37,38^. Theoretically, by increasing number of network layers, ANN generates significantly accurate results. However, increasing number of network layers is a time consuming process and makes the training process difficult to fit. Therefore, we adopted the standard classical structure of MLP that includes three-layers, one input layer; one hidden layer, and one output layer for the prediction of functional properties. Figure 1 displays the schematic structure of the proposed MLP model for this study.

There were training and testing parts in the proposed MLP model and $75\%$ of the data from Table 1 was used for the training of the proposed model whereas $25\%$ of the data was used for testing purpose respectively. Three physical factors shown in Table 1 were considered as training inputs vectors. Therefore, the number of input nodes for training was 3; the number of nodes for output layer was 4 and the number of nodes for hidden layer was calculated according to the following equation^34^:

3 $$\begin{aligned} N=\sqrt{m+n}+a, \end{aligned}$$

where *N* is the number of hidden layer nodes. *m* and *n* represent the number of input and output nodes, respectively. *a* is a constant with a value range [1, 10]. The best number of hidden layer nodes was determined from this value range.

### Model selection

The performance of the proposed MLP model was evaluated using various statistical indicators i.e., root mean squared error (RMSE), mean absolute error (MAE), Pearson correlation coefficient (*r*) and coefficient of determination ($R^{2}$), defined respectively by the following equations:

4 $$\begin{aligned} RMSE= & {} \sqrt{\frac{1}{n}\Sigma _{i=1}^{n}{(y_i-\hat{y}_i)^2}}, \end{aligned}$$

5 $$\begin{aligned} MAE= & {} \frac{1}{n}\Sigma _{i=1}^{n}\left| (y_i-\hat{y}_i)\right| , \end{aligned}$$

6 $$\begin{aligned} r= & {} \frac{ \sum _{i=1}^{n}(y_i-\bar{y})(\hat{y_i}-\bar{\hat{y}}) }{ \sqrt{\sum _{i=1}^{n}(y_i-\bar{y})^2}\sqrt{\sum _{i=1}^{n}(\hat{y_i}-\bar{\hat{y}})^2}}, \end{aligned}$$

7 $$\begin{aligned} R^{2}= & {} \left( \frac{ \sum _{i=1}^{n}(y_i-\bar{y})(\hat{y_i}-\bar{\hat{y}}) }{ \sqrt{\sum _{i=1}^{n}(y_i-\bar{y})^2}\sqrt{\sum _{i=1}^{n}(\hat{y_i}-\bar{\hat{y}})^2}}\right) ^{2}, \end{aligned}$$

where $y_i$ and $\hat{y}$ represent the actual and network outputs, respectively. *n* is the number of samples. $\bar{y}$ represents the mean of the actual variables and $\bar{\hat{y}}$ is the mean of the predicted variables.

### Sensitivity analysis

Sensitivity analysis (SA) is a statistical method that provides an idea of how sensitive is the best solution chosen to any changes in input values from one or more parameters^39^. ANOVA is an independent SA method that assesses if there is any statistically significant association between one or more inputs and output^40–43^. ANOVA utilizes the statistic ratio *F* to define if there is a significant difference exists between the average responses to main interactions or interactions between factors. The higher *F*-value indicates higher rankings. The *p*-value represents the differences between column means if they are significant or not. In this paper, one-way ANOVA is used to assesses the difference between the obtained results using the proposed MLP model, MLR and experimental values.

## Results and discussion

### Analysis of the proposed MLP model

**Figure 2:**
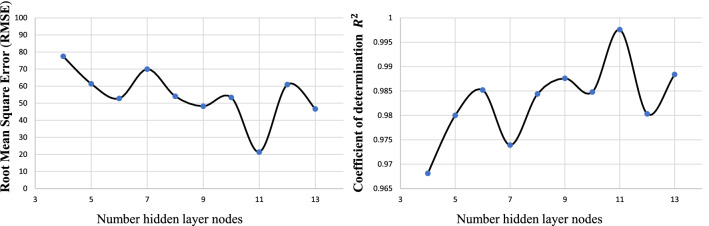
Root mean square error (RMSE) and coefficient of determination $R^{2}$ for different hidden layer nodes.

Functional properties of nano $\hbox {TiO}_2$ coated cotton fabric were predicted through backpropagation MLP model. The selected MLP model with three-layers (i.e., an input, a hidden, and an output layers) was adjusted in a way that the number of hidden layer nodes could not exceed the range of values [4, 13] according to Eq. (3). Afterwards, the results were tested for different number of hidden layer nodes in this values range. Figure 2 represents the errors and accuracy in terms of RMSE and $R^{2}$ for different number of hidden layer nodes. The obtained results reveal that the proposed MLP model with 11 nodes has the lower computation error and higher accuracy with $\hbox {RMSE}=21.3844$ and $R^{2}=0.9976$, respectively. Therefore, MLP model with 11 nodes was adopted and further used in this work.

**Figure 3:**
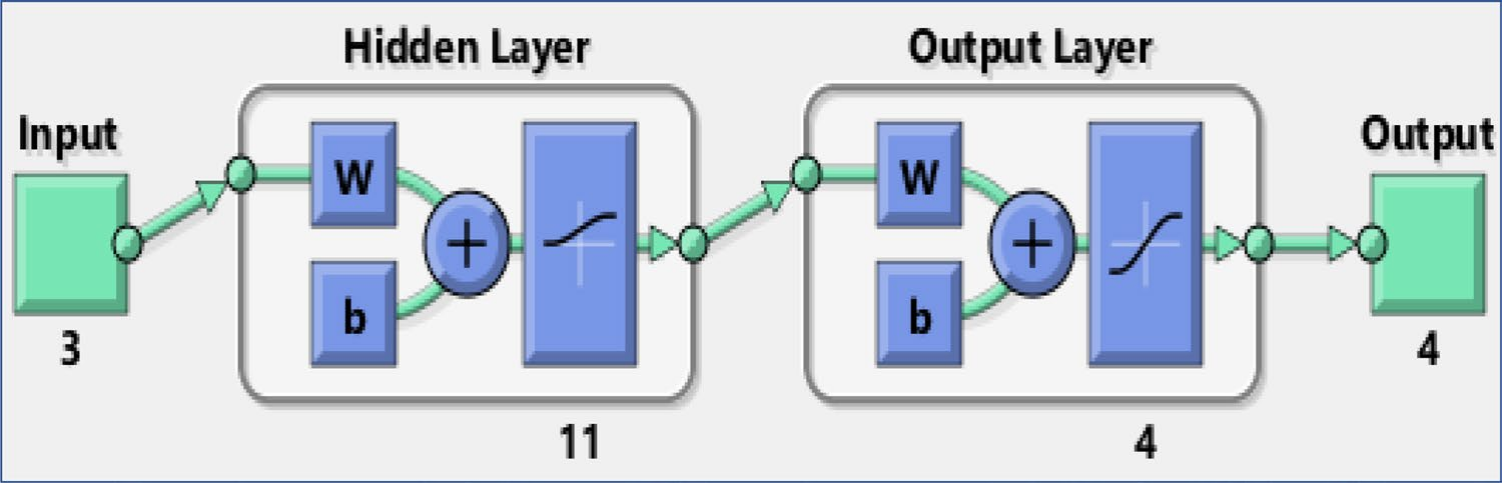
The experimental structure of MLP model.

Regarding the transfer function, there are three main transfer functions mostly used for hidden and output layers i.e., logarithmic sigmoid (logsig), tangent sigmoid (tansig) and purelin (a linear function) functions. The best selection of a transfer function for input and output layers guarantee the accuracy of the predicted results. Therefore, all possible measures were taken to assure that the tests were performed in such a way where the network structure, the thresholds and the weights were the same. Table 2 shows the RMSE, *r* and $R^{2}$ of different transfer functions. It was observed that the determination of transfer function of hidden and output layers has a significant influence on the desired prediction accuracy. The selection of logsig transfer function for hidden layer and tansig transfer function for output layer provide lower errors according to RMSE and high accuracy according to $R^{2}$ and *r*. Figure 3 represents the MLP model based on the optimal structure (number of layers and layer nodes) according to our observations and this model is further utilised for the prediction of results. The setting of training parameters are presented in Table 3.

**Table 2:** Errors of different transfer functions.

Hidden layer function	Output layer function	$R^{2}$	*r*	RMSE
Purelin	Purelin	0.9796	0.9897	61.9663
Purelin	Tansig	0.9740	0.9869	69.8786
Purelin	Logsig	0.8576	0.9261	163.5759
Logsig	Tansig	0.9976	0.9988	21.3844
Logsig	Logsig	0.8469	0.9203	169.5826
Logsig	Purelin	0.9788	0.9893	63.0491
Tansig	Tansig	0.9829	0.9914	56.5975
Tansig	Logsig	0.8337	0.9131	176.7290
Tansig	Purelin	0.9871	0.9935	49.2292

**Table 3:** Parameters and settings of training network.

Parameters	Settings
Training function	Trainbr
Transfer function of Hidden layer	Logsig
Transfer function of Output layer	Tansig
Epochs	1000
Input node	3
Hidden node	11
Output node	4
Learning rate	0.02
Performance goal	0.00001

### MLR model

The purpose of MLR is to investigate the relationship between independent variables i.e., (amount of titanium tetrachloride, amount of isopropanol, and sonication time) and the obtained results i.e., (self-cleaning efficiency, antimicrobial efficiency and UPF) for nano $\hbox {TiO}_2$ coated cotton fabric. Moreover, the performance of MLR and MLP were compared using the MAE, RMSE, correlation coefficient and the coefficient of determination released in the four properties.

### Comparison of MLP and MLR results

**Figure 4:**
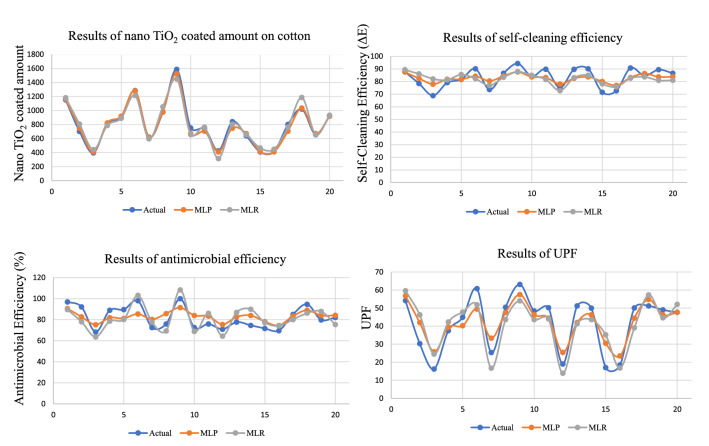
The predicted and actual values using MLP and MLR.

**Figure 5:**
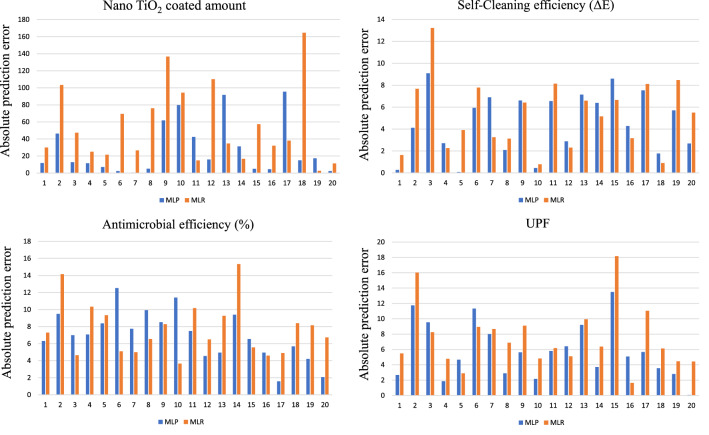
Absolute errors between predicted and actual values using MLP and MLR.

A comparison of the predicted results of MLP model was made with MLR results of all functional properties for different amount of precursor and solvent under varying reaction time. The predicted results for all four outputs are presented in Fig. 4. The absolute errors given by the difference between predicted and actual values for both MLP and MLR is shown in Fig. 5.

The cumulative mean of both RMSE and MAE, $R^{2}$, and *r* of both MLP and MLR models for all four functional properties are presented in Table 4. The results reveal that the values of estimation error were significantly lower for the proposed MLP model as compared to MLR for all four functional properties.

**Table 4:** The performance measures of MLP and MLR for functional properties of nano $\hbox {TiO}_2$ coated cotton.

Multifunctional properties	Methods	RMSE	MAE	$\hbox {R}^{2}$	*r*
Nano $\hbox {TiO}_2$ Coated amount	MLP	41.19	27.97	0.903	0.9503
	MLR	70.94	55.64	0.86	0.9274
Self-cleaning efficiency	MLP	5.35	4.59	0.95	0.9747
	MLR	6.10	5.25	0.52	0.7211
Antimicrobial efficiency	MLP	7.51	6.98	0.95	0.9747
	MLR	8.27	7.69	0.54	0.7348
UPF	MLP	6.82	5.81	0.93	0.9644
	MLR	3.45	7.46	0.85	0.9220

**Figure 6:**
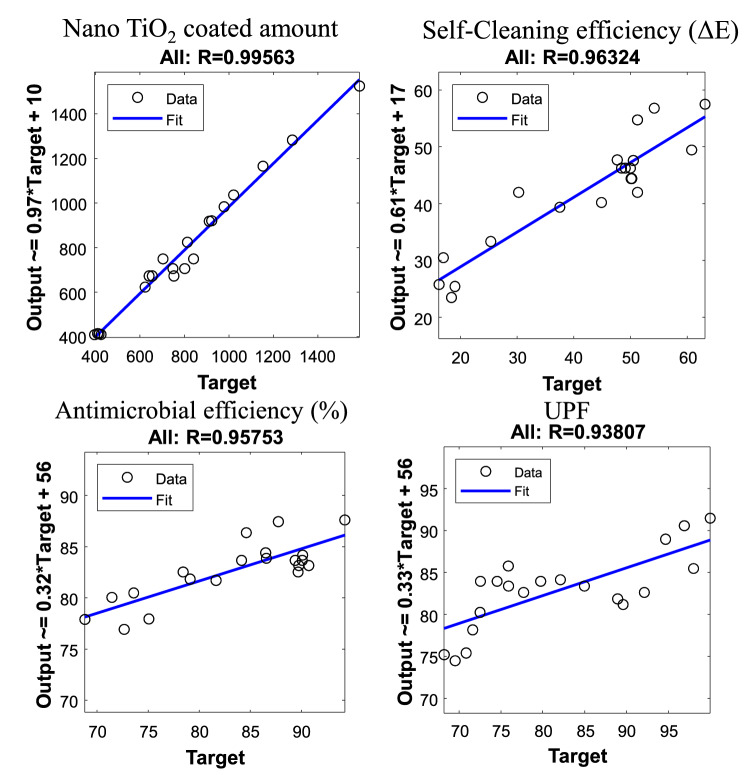
Correlation between the predicted and actual values for overall sets of data using MLP model.

The correlation between the actual and the predicted values using MLP of all tested properties is illustrated in Fig. 6. It is observed from the results that the correlation ($R-$value) provide a very strong relationship between the actual and predicted values, where $R = 99\%$ for Nano $\hbox {TiO}_2$ coated amount, $R = 96 \%$ for Self-Cleaning efficiency, $R = 95 \%$ for antimicrobial efficiency, and $R = 93\%$ for UPF.

**Figure 7:**
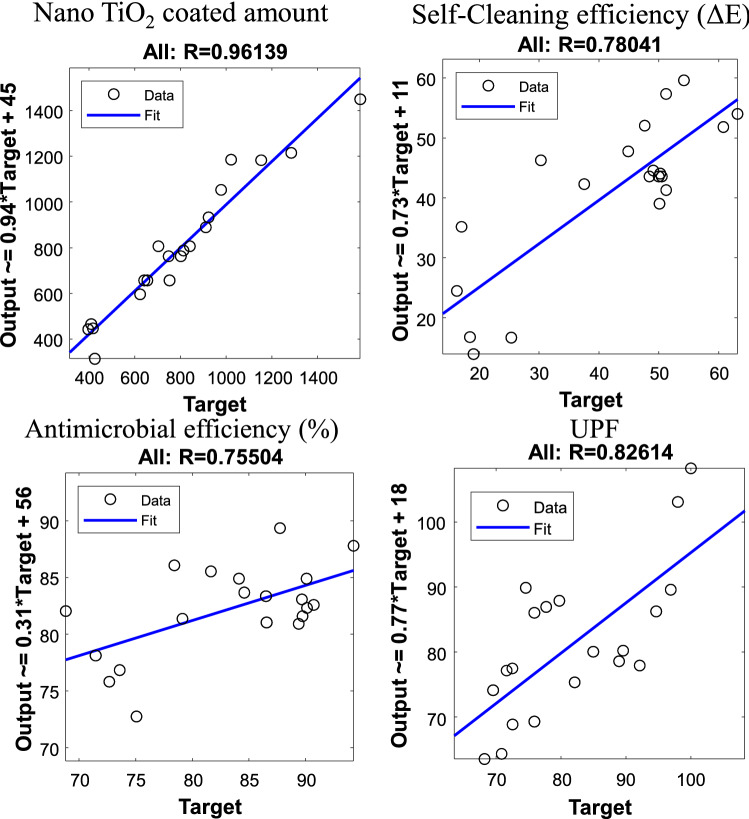
Correlation between the predicted and actual values using MLR model.

The relation between actual and predicted values by MLR model had been plotted using a linear regression model in Fig. 7. We noted that MLR model provide a good correlation between the actual and predicted values especially for the first functional property Nano $\hbox {TiO}_2$ coated amount, where $R = 96\%$. However, the results shows less accuracy for the other three functionals properties, where $R = 78 \%$ for Self-Cleaning efficiency, $R = 75 \%$ for antimicrobial efficiency, and $R = 82\%$ for UPF. As result, MLP outperformed MLR for the prediction of the all functional properties.

### Robustness assessment

A one-way ANOVA test was applied to assess the robustness of the predicted results using MLP, MLR as well as the experiment data. ANOVA test helps to knows how it is the correlations between the predicted responses of coated fabric with process variables. Table 5 shows the results of the one-way ANOVA test of each functional properties obtained by MLP, MLR and experimental. It is observed that the developed MLP model was more statistically significant as compared to MLR and experimental, as it provides the lowest $p$-value for all functional properties.

**Table 5:** Analysis report of the experimental values and the predicted values using MLP and MLR for functional properties of nano $\hbox {TiO}_2$ coated cotton.

Multifunctional properties	Methods	p-value	F-value
Nano $\hbox {TiO}_2$ coated amount	Experimental	8.38e−7	29.99
	MLP	3.24e−8	47.88
	MLR	4.22e−7	33.18
Self-cleaning efficiency	Experimental	0.001	8.92
	MLP	7.17e−11	109.38
	MLR	0.007	5.78
Antimicrobial efficiency	Experimental	2.65e−5	17.47
	MLP	2.18e−11	127.76
	MLR	0.005	6.284
UPF	Experimental	2.33e−5	17.86
	MLP	7.61e−10	79.51
	MLR	6.12e−7	31.42

## Conclusions

In this paper, a specific class of ANN called MLP model was developed and compared with MLR for the prediction of functional properties of nano $\hbox {TiO}_2$ coated cotton fabric. The developed MLP model was used to correlate different output results with varying input variables. The obtained results showed a higher prediction accuracy for the developed MLP model i.e., ($p<0.1 e^{-10}$ and $r>95\%$) compared to MLR. The computed values of RMSE and MAE from all predicted results confirmed that the proposed MLP model has lower error as compared to MLR. The successful utilization of the developed model revealed a non-linear relationship between the selected parameters for the prediction of functional properties. The findings of this work highlight that MLP approach can be effectively used for the prediction of other properties of nano coated fabrics.
